# Arginase-1–Expressing Macrophages Suppress Th2 Cytokine–Driven Inflammation and Fibrosis

**DOI:** 10.1371/journal.ppat.1000371

**Published:** 2009-04-10

**Authors:** John T. Pesce, Thirumalai R. Ramalingam, Margaret M. Mentink-Kane, Mark S. Wilson, Karim C. El Kasmi, Amber M. Smith, Robert W. Thompson, Allen W. Cheever, Peter J. Murray, Thomas A. Wynn

**Affiliations:** 1 Laboratory of Parasitic Diseases, National Institute of Allergy and Infectious Diseases, National Institutes of Health, Bethesda, Maryland, United States of America; 2 Departments of Infectious Diseases and Immunology, St. Jude Children's Research Hospital, Memphis, Tennessee, United States of America; 3 Biomedical Research Institute, Rockville, Maryland, United States of America; Case Western Reserve University, United States of America

## Abstract

Macrophage-specific expression of Arginase-1 is commonly believed to promote inflammation, fibrosis, and wound healing by enhancing L-proline, polyamine, and Th2 cytokine production. Here, however, we show that macrophage-specific Arg1 functions as an inhibitor of inflammation and fibrosis following infection with the Th2-inducing pathogen *Schistosoma mansoni*. Although susceptibility to infection was not affected by the conditional deletion of *Arg1* in macrophages, *Arg1* ^−/flox^ *;LysMcre* mice died at an accelerated rate. The mortality was not due to acute Th1/NOS2-mediated hepatotoxicity or endotoxemia. Instead, granulomatous inflammation, liver fibrosis, and portal hypertension increased in infected *Arg1* ^−/flox^ *;LysMcre* mice. Similar findings were obtained with *Arg1* ^flox/flox^ *;Tie2cre* mice, which delete *Arg1* in all macrophage populations. Production of Th2 cytokines increased in the infected *Arg1* ^−/flox^ *;LysMcre* mice, and unlike alternatively activated wild-type macrophages, *Arg1* ^−/flox^ *;LysMcre* macrophages failed to inhibit T cell proliferation *in vitro*, providing an underlying mechanism for the exacerbated Th2 pathology. The suppressive activity of Arg1-expressing macrophages was independent of IL-10 and TGF-β1. However, when exogenous L-arginine was provided, T cell proliferation was restored, suggesting that Arg1-expressing macrophages deplete arginine, which is required to sustain CD4^+^ T cell responses. These data identify Arg1 as the essential suppressive mediator of alternatively activated macrophages (AAM) and demonstrate that Arg1-expressing macrophages function as suppressors rather than inducers of Th2-dependent inflammation and fibrosis.

## Author Summary

While the function of NOS2 in Th1-type immunity has been investigated extensively, the role of Arg1 in the regulation of Th2-type responses is unclear. Previously, we showed that proline production in AAMs is regulated by Arg1 activity. Because proline is essential for collagen synthesis in myofibroblasts, numerous studies have suggested that Arg1-expressing AAMs regulate wound healing and fibrosis. The development of fibrosis in schistosomiasis is dependent on Th2 cytokines, and mice deficient in IL-4/IL-13 fail to upregulate Arg1. Nevertheless, although Arg1 expression is associated with Th2-dependent fibrosis, the contribution of macrophage-specific Arg1 to the pathogenesis of fibrosis in schistosomiasis was unknown. The studies conducted here with two different strains of mice deficient in macrophage-associated Arg1 demonstrate unequivocally that Arg1-expressing macrophages exhibit both anti-inflammatory and anti-fibrotic activity during Th2-driven inflammatory responses. In schistosomiasis, fibrosis, portal hypertension, and variceal bleeding are the primary pathological changes that characterize the severe hepatosplenic form of the disease in humans. It is widely believed that people who fail to adequately activate immune suppressive mechanisms when chronically infected with *S. mansoni* are the individuals who ultimately develop severe disease. Our data identify Arg1-expressing macrophages as critical mediators of immune downmodulation in chronic schistosomiasis.

## Introduction

Arginase 1 (Arg1) is a cytosolic enzyme constitutively expressed in the liver where it functions in nitrogen elimination by catalyzing arginine hydrolysis to urea and ornithine [1],[2]. Arg1 is also expressed in macrophages but unlike the constitutive expression observed in the liver, *Arg1* gene expression is tightly regulated by exogenous stimuli including the Th2 cytokines IL-4 and IL-13 [3],[4]. The production of urea removes excess nitrogen from the body, while ornithine can be used to generate polyamines, glutamate, and proline, the latter of which is critical to the synthesis of collagen [5],[6]. Arg1 also competes with iNOS (encoded by *Nos2*) an enzyme that controls the production of nitric oxide in IFN-γ stimulated classically activated macrophages (CAMs). Thus, expression of *Nos2* and *Arg1* currently defines classically and alternatively activated macrophages (AAMs), respectively. Nevertheless, while iNOS and Arg1 have been studied in the context of Th1 responses [7],[8], the role of *Arg1* in Th2-polarized immune responses remains unknown.

Mice infected with the parasitic trematode *Schistosoma mansoni* are a model of human schistosomiasis and chronic Th2 cytokine-mediated pathology, as many of the pathological features of the human diseases are duplicated in the mouse model. *S. mansoni* infection leads to the development of a Th2-driven inflammatory response in response to the eggs deposited in the intestines and liver by adult worm pairs. The resulting immune response is driven by the production of Th2 cytokines including IL-4, IL-5, IL-13, and IL-21, which contribute to the development of hepatic fibrosis and portal hypertension [2],[9],[10]. The Th2 response is also associated with the accumulation of a large number of Arg1-expressing macrophages in and around the granulomas [10],[11]. Because Arg1 regulates the synthesis of the amino acid proline, which is required for collagen production, numerous studies have suggested that Arg1 is critically involved in the regulation of wound healing and fibrosis [2],[11],[12]. However, the specific function of Arg1 remains unexplored. A few recent studies have begun to dissect the function of arginases in immunity by using RNA interference, macrophage depletion, and the arginase inhibitor *S*-(2-boronoethyl)-l-cysteine (BEC) [13]–[15]. However, these approaches are not specific to the Arg1 isoform nor are they selective antagonists of Arg1 in macrophages. Because *Arg1* mRNA and Arg1 protein is predominantly produced by macrophages following infection with *S. mansoni* [11], we examined whether Arg1 regulates the pathogenesis of Th2 cytokine driven disease by studying mice with macrophage-specific deletions of *Arg1*.

## Results

### Generation and characterization of macrophage-specific *Arg1* null mice

Mice in which a portion of the *Arg1* gene was flanked with loxP sites were crossed with mice expressing the Cre recombinase from the *lysozyme M* (*LysM*) gene (Lyz2^tm1(cre)Ifo^), hereafter referred to as ‘*LysMcre*’ to generate mice in which *Arg1* was deleted in macrophages [16]. It is important to note that although *LysM* expression has been observed in several tissues as well as in the later steps of myeloid lineage differentiation [17],[18], inducible extra-hepatic *Arg1* expression is predominantly found in macrophages and possibly dendritic cells as recently shown by a IRES-YFP knock-in allele that reads out Arg1 expression [19]. Therefore, our system tests Arg1 function in macrophages, because these are the main cells that express Arg1, regardless of the broader expression of the cre deleter used.

**Figure 1:**
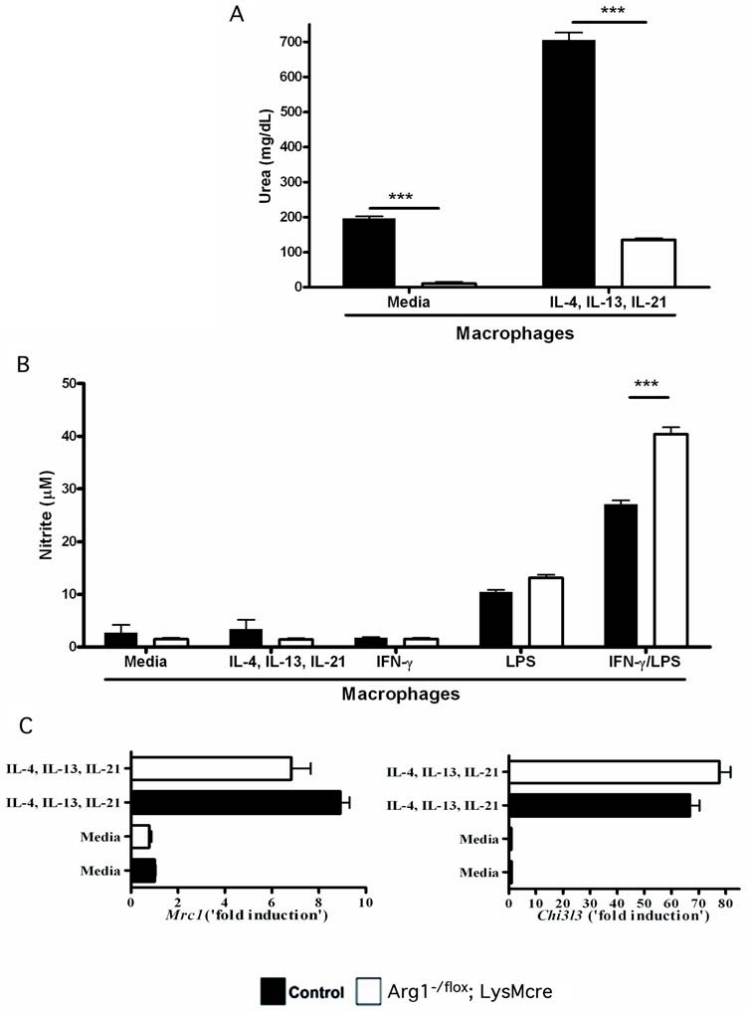
*Arg1* ^−/flox^ *;LysMcre* mice exhibit reduced Arg1 activity in macrophages. (A) Macrophages were isolated from control (filled bars) and *Arg1* ^−/flox^ *;LysMcre* (open bars) mice and analyzed for arginase activity by measuring urea. Macrophages were isolated and assayed with either medium alone or treated with a combination of IL-4, IL-13, and IL-21 overnight. (B) Macrophages were assayed for NO by Griess reaction in the presence of medium, IL-4, IL-13, and IL-21, or IFN-γ and LPS (Mean±SEM). (C) RNA from thioglycollate elicited macrophages stimulated with IL-4, IL-13, and IL-21 was isolated, purified, and assessed for the expression of *Mrc1* (mannose receptor) and *Chi3l3* (Ym1) (Mean±SEM). All assays were repeated with similar results and performed in triplicate.

As a result, thioglycollate-elicited macrophages isolated from *Arg1* ^−/flox^ *;LysMcre* mice showed markedly reduced arginase activity when examined ex vivo (Figure 1A). *Arg1* ^−/flox^ *;LysMcre* macrophages also showed a significant reduction in arginase activity following stimulation with the Th2 cytokines IL-4, IL-13, and IL-21 [10] (approximately 7-fold decrease from Arg1^flox/flox^ controls). In contrast, NO production remained intact (Figure 1B), and was slightly elevated following IFN-γ/LPS stimulation. The Arg1-deficiency in macrophages did not alter the expression of other markers of alternative activation since thioglycolate-elicited macrophages from both *Arg1* ^flox/flox^ and *Arg1* ^−/flox^ *;LysMcre* mice displayed similar increases in *Mrc1* (mannose receptor) and *Chi3l3* (Ym1) gene expression following stimulation with IL-4, IL-13, and IL-21 (Figure 1C). Therefore, Arg1 is not involved in the development of AAMs but is critically required for Th2 cytokine-inducible arginase activity in macrophages.

### Macrophage-specific Arg1 suppresses the development of fibrosis

**Figure 2:**
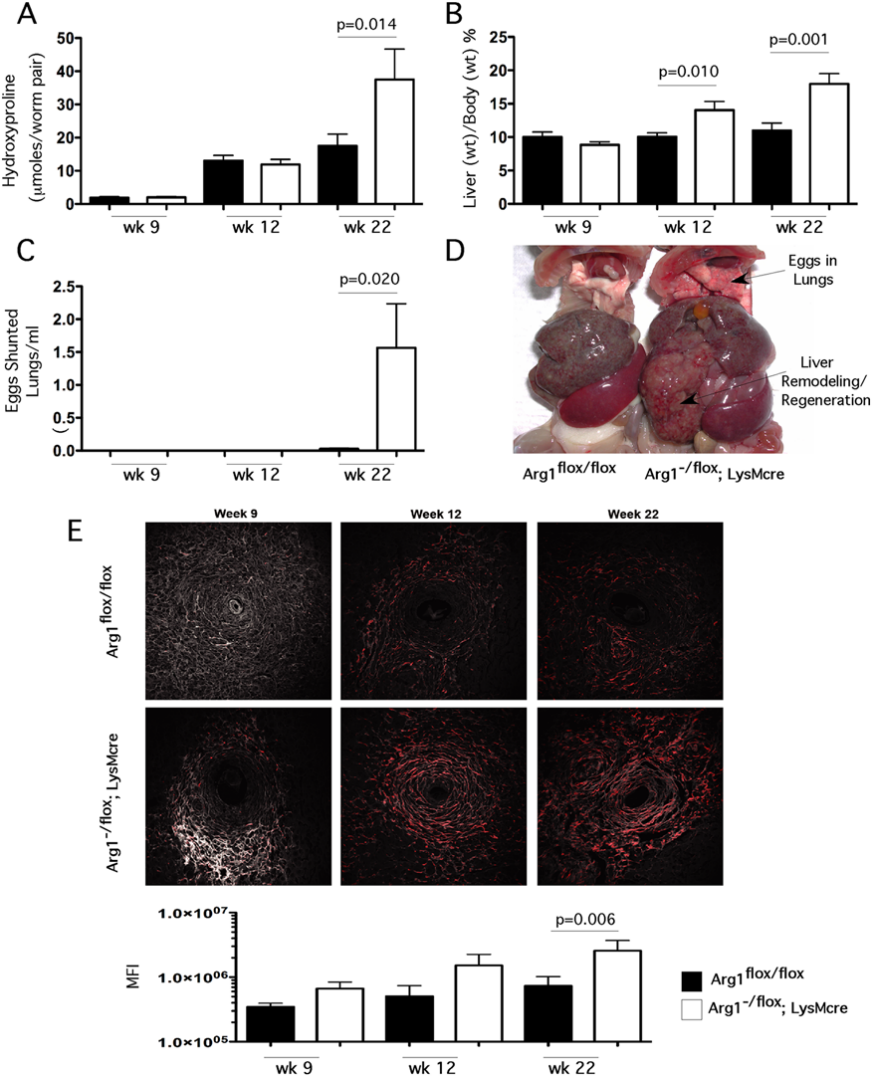
*Arg1* expression is required to control fibrosis. Control *Arg1* ^flox/flox^ (filled bars) and *Arg1* ^−/flox^ *;LysMcre* (open bars) mice were exposed to 35 *S. mansoni* cercariae percutaneously. Mice (*n* = control/*Arg1* ^−/flox^ *;LysMcre* ) were sacrificed at weeks 9 (*n* = 14/14), 12 (*n* = 21/10), and 22 (*n* = 12/10) post-infection and analyzed for the development of fibrosis and portal hypertension. (A) Collagen was assessed by measuring hydroxyproline and normalizing to infectious worm pairs per mouse (Mean±SEM). (B) Total animal weight was compared with the weight of total excised liver to determine liver as a percent of body weight (Mean±SEM). (C) *S. mansoni* eggs within the lungs of control and *Arg1* ^−/flox^ *;LysMcre* mice as an measure of collateral vessel development and portal hypertension; eggs were enumerated by digesting lungs in 4% KOH at 37°C for 12 hours and 1ml of the suspension was counted in a Sedgwick-Rafter chamber (Mean±SEM). (D) Representative gross pathology of 22-week infected control and *Arg1* ^−/flox^ *;LysMcre.* Upper arrow indicates presence of *S. mansoni* eggs which were shunted into the lungs of *Arg1* ^−/flox^ *;LysMcre* mice. (E) Individual liver sections from Control *Arg1* ^flox/flox^ (top) and *Arg1* ^−/flox^ *;LysMcre* (bottom) mice were analyzed for collagen via second harmonic emission (red). Representative granulomas from 9, 12, and 22 weeks post-infection with *S. mansoni.* All images were taken at 20× magnification. Mean fluorescence intensities for individual control (filled bars) and *Arg1* ^−/flox^ *;LysMcre* (open bars) mice at week 9 (*n* = 14/14), 12 (*n* = 21/10), and 22 (*n* = 12/9) weeks post-infection with *S. mansoni.* All assays were repeated three times with similar results.

To determine whether macrophage specific *Arg1* regulates tissue fibrogenesis, control mice (*Arg1* ^flox/flox^ *)* and *Arg1* ^−/flox^ *;LysMcre* litter mates were percutaneously exposed to 35 *S. mansoni* cercariae and the progressive development of fibrosis was assessed in the liver at 9 (acute), 12 (early chronic), and 22 wk (late chronic) post-infection by measuring hydroxyproline, a modified amino acid found in collagen. Infectious burdens, assessed by adult worm pairs and tissue egg counts in the liver and gut, were similar in both *Arg1* ^flox/flox^ and *Arg1* ^−/flox^ *;LysMcre* mice at all time points, demonstrating that *Arg1*-deficiency does not affect susceptibility to infection (Table S1). In addition, several genes that characterize the development of AAMs, including *Chi3l3* (Ym1) and *Retnla* (Relm-alpha/FIZZ1), were increased similarly in the livers of both groups (data not shown), which was consistent with our in vitro studies with Arg1-deficient macrophages (Figure 1). Although Arg1 was hypothesized to be critical for collagen deposition [1],[2], the *Arg1* ^−/flox^ *;LysMcre* mice showed no impairment in hepatic fibrosis at the acute stage of infection, and surprisingly, displayed marked and highly significant increases in fibrosis when chronically infected (Figure 2A). Similar findings were obtained using second harmonic confocal microscopy, which can also be used to quantify the collagen content of liver sections (Figure 2E). The livers of the infected *Arg1*-deficient mice were also significantly enlarged at 12 and 22 weeks, providing evidence of extensive remodeling and regeneration of the liver (Figure 2B and 2D). The marked increase in hepatic fibrosis in the *Arg1* ^−/flox^ *;LysMcre* mice was also associated with enhanced shunting of eggs to the lungs (Figure 2C), providing evidence of increased portal hypertension and development of porto-systemic shunts [9], complications commonly associated with chronic liver fibrosis. Thus, the *Arg1* ^−/flox^ *;LysMcre* mice displayed several pathological features that characterize the severe hepatosplenic form of schistosomiasis. These data demonstrate that macrophage-specific *Arg1* expression is not required for the development of Th2 cytokine dependent fibrosis [2],[12]. Instead, the conditional deletion of *Arg1* in macrophages caused a significant exacerbation of schistosomiasis.

### Arg1 downregulates chronic inflammation

**Figure 3:**
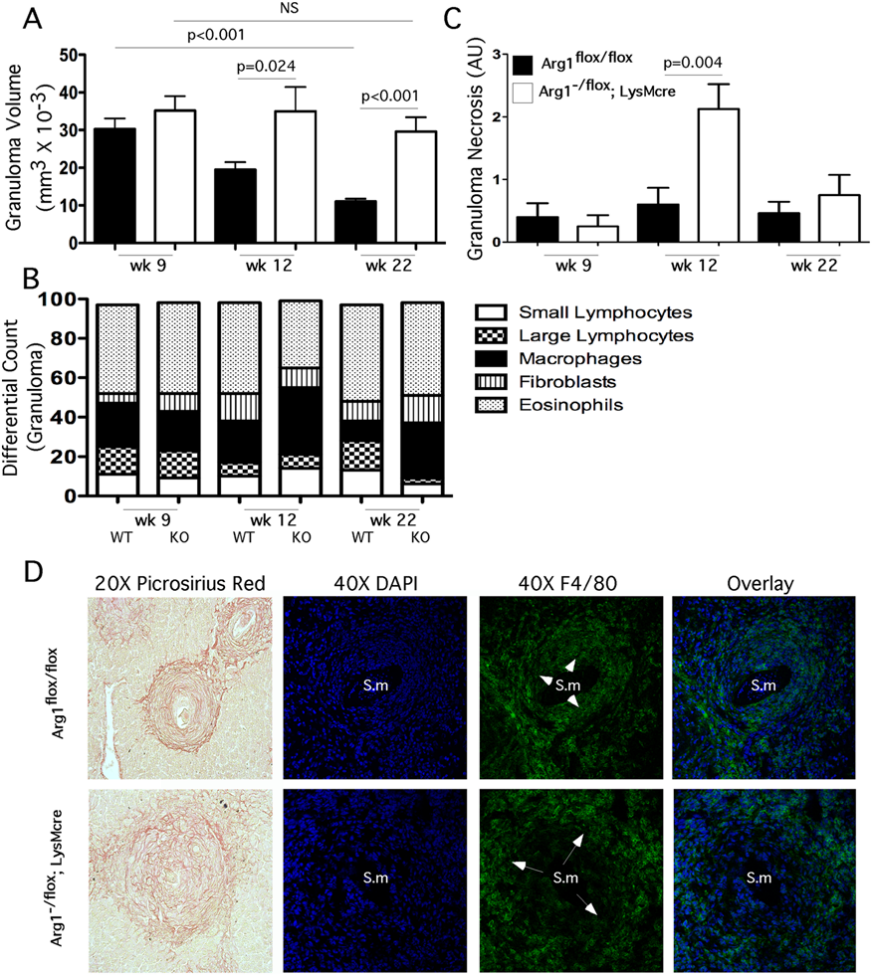
Macrophage-associated Arg1 inhibits granulomatous inflammation. Week 9 (*n* = 14/14), 12 (*n* = 21/10), and 22 (*n* = 12/9) *S. mansoni*–infected control and *Arg1* ^−/flox^ *;LysMcre* mice were individually assessed for granuloma volume (Mean±SEM) (A), and individual populations of small/large lymphocytes, macrophages, fibroblasts, and eosinophils were enumerated (B). Individual granulomas from control (filled bars) and *Arg1* ^−/flox^ *;LysMcre* mice (open bars) were scored for granuloma-associated necrosis on a scale of 1–4 at weeks 9 (*n* = 14/14), 12 (*n* = 21/10) and 22 (*n* = 12/9) post-infection with *S. mansoni* (mean±SEM) (C). Representative granulomas from 12-week *S. mansoni*–infected control and *Arg1* ^−/flox^ *;LysMcre* mice were stained with Picrosirius Red 20× or DAPI (blue) and F4/80^+^ (green) and photographed at 40×. Arrows point to macrophage-rich regions. Sm = *S. mansoni* egg (D). All assays were repeated three times with similar results.

Although chronic inflammation typically contributes to tissue remodeling and fibrosis, recent studies have suggested that the mechanisms controlling inflammation and fibrosis are distinct [20]. In addition to regulating proline production in macrophages [11], Arg1 is also involved in the generation of polyamines, which are important regulators of cellular proliferation [6],[21]. Therefore, to determine whether the development of hepatomegaly and fibrosis in the infected *Arg1* ^−/flox^ *;LysMcre* mice was associated with changes in inflammation, histological analyses were performed on liver sections to quantify both granuloma size and the proportion of various leukocytic cell types within individual granulomas. As predicted, granuloma size in the *Arg1* ^flox/flox^ control mice peaked on wk 9 and subsequently decreased on wks 12 and 22 (Figure 3A). In contrast to the control mice, however, the infected *Arg1* ^−/flox^ *;LysMcre* mice failed to downmodulate their granulomatous inflammatory response. Instead, their liver granulomas were nearly 3-fold larger than controls at the chronic stage of infection and remained of comparable size to the lesions found in acutely infected (wk 9) *Arg1* ^−/flox^ *;LysMcre* mice. A similar increase in granuloma size was also found in the small intestines of *Arg1* ^−/flox^ *;LysMcre* mice (Figure S1). Similar to the liver, inflammation in the gut was limited to the granulomas with no diffuse parenchymal component. There was also no unusual histologic pathology in the gut as was reported in *S. mansoni* infected *Il4ra* ^−/flox^ *;LysMcre* and *Il4* ^−/−^ mice [22],[23]. Despite developing larger lesions, the cellular composition of the *Arg1* ^−/flox^ *;LysMcre* granulomas were similar to controls, with macrophages and eosinophils predominating in both cases (Figure 3B). A key difference noted in the *Arg1* ^−/flox^ *;LysMcre* granulomas, however, was a consistent increase in the number of macrophages, particularly in the chronically infected animals (Figure 3B). Confocal fluorescent microscopy revealed a small ring of F4/80^+^ macrophages in close proximity to the eggs in the control *Arg1* ^flox/flox^ mice, while a much larger band of macrophages was found at the periphery of the *Arg1* ^−/flox^ *;LysMcre* granulomas (Figure 3D). Granuloma-associated liver necrosis was also significantly increased in *Arg1* ^−/flox^ *;LysMcre* mice (Figure 3C), most notably at the 12 wk chronic time point, although in experiments with increased infectious burdens, significant necrosis was observed in the *Arg1* ^−/flox^ *;LysMcre* mice as early as 9 wk post-infection (WT: 0.14±0.14, KO: 2.71±0.61, p<0.001).

### Inflammation and fibrosis are exacerbated in Arg1^flox/flox^;*Tie2cre* mice

**Figure 4:**
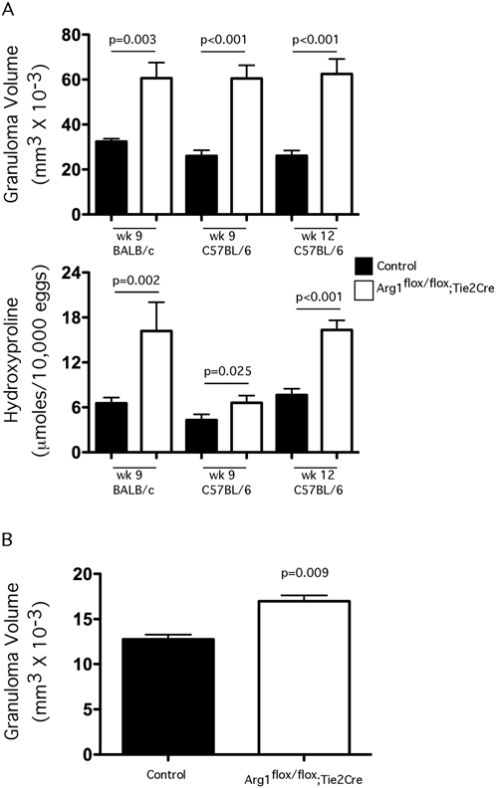
Arg1^flox/flox^;Tie2cre mice develop exacerbated inflammation and fibrosis. (A) BALB/c control (*n* = 5), BALB/c *Arg1* ^flox/flox^ *;Tie2cre* (*n* = 5), C57BL/6 control (*n* = 5), and C57BL/6 *Arg1* ^flox/flox^ *;Tie2cre* (*n* = 7) were infected with *S. mansoni* cercariae and euthanized on week 9 and assessed individually for granuloma volume and fibrosis. Additional control (*n* = 6) and C57BL/6 *Arg1* ^flox/flox^ *;Tie2cre* (*n* = 5) were examined on week 12. The data show the means±SEM; significant differences are noted in the figure. (B) Granuloma volumes were measured in the intestines of control and C57BL/6 *Arg1* ^flox/flox^ *;Tie2cre* on week 12 (Mean±SEM). A second experiment produced similar results.

We also generated a second mouse strain lacking Arg1 in macrophages using the *Tie2cre* deleter mouse (B6.Cg-*Tg(Tek-cre)12Flv*/J). Although the Tie2cre mouse can delete floxed genes in all hematopoietic and endothelial lineage cells, because Arg1 is expressed in myeloid but not lymphoid lineage cells [19],[24], the different strains provided parallel systems for specifically testing the function of Arg1 in macrophages *in vivo*. The *Arg1* conditional knockouts were crossed to *Tie2cre* mice and backcrossed on both the BALB/c and C57BL/6 genetic backgrounds so they also provided a tool to examine the influence of Arg1 activity on two distinct backgrounds. Macrophages isolated from the *Tie2cre* cross were previously shown to develop a near complete ablation of Arg1 enzymatic activity in all macrophage types tested (>99%), in contrast to the Arg1^−/flox^;LysMcre mice, which deleted approximately >85–90% of arginase activity in macrophages [8]. We used BALB/c and C57BL/6 littermate control *Arg1* ^flox/flox^ and *Arg1* ^flox/flox^;*Tie2cre* mice infected with 35 *S. mansoni* cercariae and inflammation and fibrosis and examined in the liver (Figure 4A) on wk 9 (BALB/c and C57BL/6) and wk 12 (C57BL/6) post-infection. As we observed with the *Arg1* ^−/flox^ *;LysMcre* mice, the *Arg1* ^flox/flox^;*Tie2cre* mice developed significantly larger granulomas at both time points. Similar results were obtained on both genetic backgrounds. An increase in granulomatous inflammation was also observed in the intestines of C57BL/6 *Arg1* ^flox/flox^;*Tie2cre* mice (Figure 4B). When hydroxyproline was measured in liver, however, the BALB/c *Arg1* ^flox/flox^;*Tie2cre* mice showed a much more striking and significant increase in fibrosis on wk 9 when compared with 9 wk infected C57BL/6 *Arg1* ^flox/flox^;*Tie2cre* mice (Figure 4A, lower panel). Nevertheless, by wk 12, the C57BL/6 *Arg1* ^flox/flox^;*Tie2cre* also displayed a notable increase in fibrosis. As such, these data suggest that macrophages are the critical cell type producing Arg1 in *S. mansoni* infection because the *LysMcre* and *Tie2cre* mice were remarkably concordant.

### Arg1 protects mice from the lethal effects of Th2 pathology

**Figure 5:**
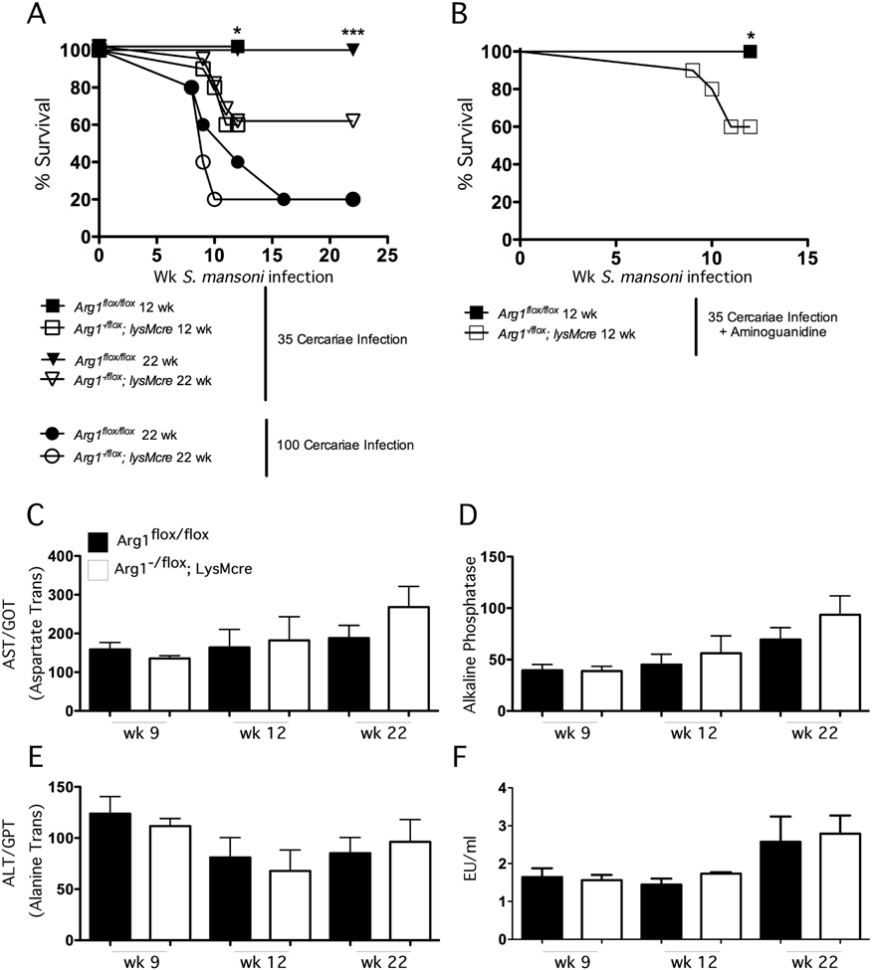
*Arg1*-expressing AAMs regulate survival after *S. mansoni* infection. Control *Arg1* ^flox/flox^ and *Arg1* ^−/flox^ *;LysMcre* mice were infected with *S. mansoni* for 12 or 22 weeks, and survival was monitored. (A) Survival rates for week 12 (*n* = 21/20) and 22 (*n* = 12/15) control and *Arg1* ^−/flox^ *;LysMcre* mice infected with 35 cercariae or week 22 mice (*n* = 5/5) infected with 100 cercariae. (B) Survival of control (*n* = 8) and *Arg1* ^−/flox^ *;LysMcre* (*n* = 9) mice infected with 35 cercariae in the presence of the NO inhibitor aminoguanidine (2.5% in drinking water). Individual serum samples were isolated from control and *Arg1* ^−/flox^ *;LysMcre* mice infected with *S. mansoni* for 9, 12, and 22 weeks. Samples were analyzed for liver enzymes and LPS. (C) AST/GOT. (D) Alkaline Phosphatase. (E) ALT/GPT. (F) Endotoxin. All assays were repeated three times with similar results.

To test whether IL-4/IL-13-induced arginase activity in macrophages was critical to the development of morbidity and mortality in schistosomiasis, we analyzed the survival of *Arg1* ^flox/flox^ and *Arg1* ^−/flox^ *;LysMcre* mice following infection with *S. mansoni* cercariae. Egg deposition in the liver commences approximately 4 wk post-infection, with peak granuloma-associated inflammation occurring by wk 8. However, despite vigorous inflammatory responses, both groups showed little to no mortality through wk 9 of infection. Nevertheless, as the infection progressed to the chronic stage, a significant difference in survival was observed between the *Arg1* ^flox/flox^ and *Arg1* ^−/flox^ *;LysMcre* mice, with ∼40% of the *Arg1* ^−/flox^ *;LysMcre* mice succumbing to the infection by wk 12 (Figure 5A). No mortality was observed in the *Arg1* ^flox/flox^ control group during this period. Additional studies were also performed with a dose of parasites that induces mortality in WT animals. Here, the *Arg1* ^−/flox^ *;LysMcre* mice also displayed an accelerated time to death, with only 20% of the *Arg1*-deficient mice surviving beyond wk 10. In contrast, only 50% of the *Arg1* ^flox/flox^ mice died by wk 12 (Figure 5A). To dissect the possible mechanisms for the increased mortality, we examined whether iNOS activity was involved. For these studies, the *Arg1* ^−/flox^ *;LysMcre* mice were treated with aminoguanidine to inhibit iNOS, as Arg1 activity is thought to potently antagonize NO production [3],[25]. Although the dose of aminoguanidine we used is highly effective at blocking NOS2 activity in vivo [26],[27], the inhibitor had no effect on the survival of the *Arg1* ^−/flox^ *;LysMcre* mice (Figure 4B). We also examined the serum levels of LPS and determined that endotoxin was not significantly increased in either group (Figure 5F), indicating that there was no disruption in gut barrier function as has been reported in *S. mansoni* infected *Il4* ^−/−^ and *Il4ra* ^−/flox^;*LysMcre* mice [23],[28],[29]. Finally, while both groups displayed significant increases in serum AST, ALT, and alkaline phosphatase following infection, the amounts in *Arg1* ^−/flox^ *;LysMcre* mice were comparable to control mice at all time points (Figure 5C–5E). Together, these observations suggest that the cause of death in the *Arg1* ^−/flox^ *;LysMcre* mice is distinct from the mechanism observed in *Il4* ^−/−^ and *Il4ra* ^−/flox^;*LysMcre* mice [23],[28], which was attributed to acute Th1 cytokine and endotoxin-mediated toxicity. Instead, we frequently observed blood in the intestine of the infected *Arg1* ^−/flox^ *;LysMcre* mice at autopsy.

### *Arg1*-expressing AAMs inhibit CD4^+^ T cell proliferation and cytokine production

**Figure 6:**
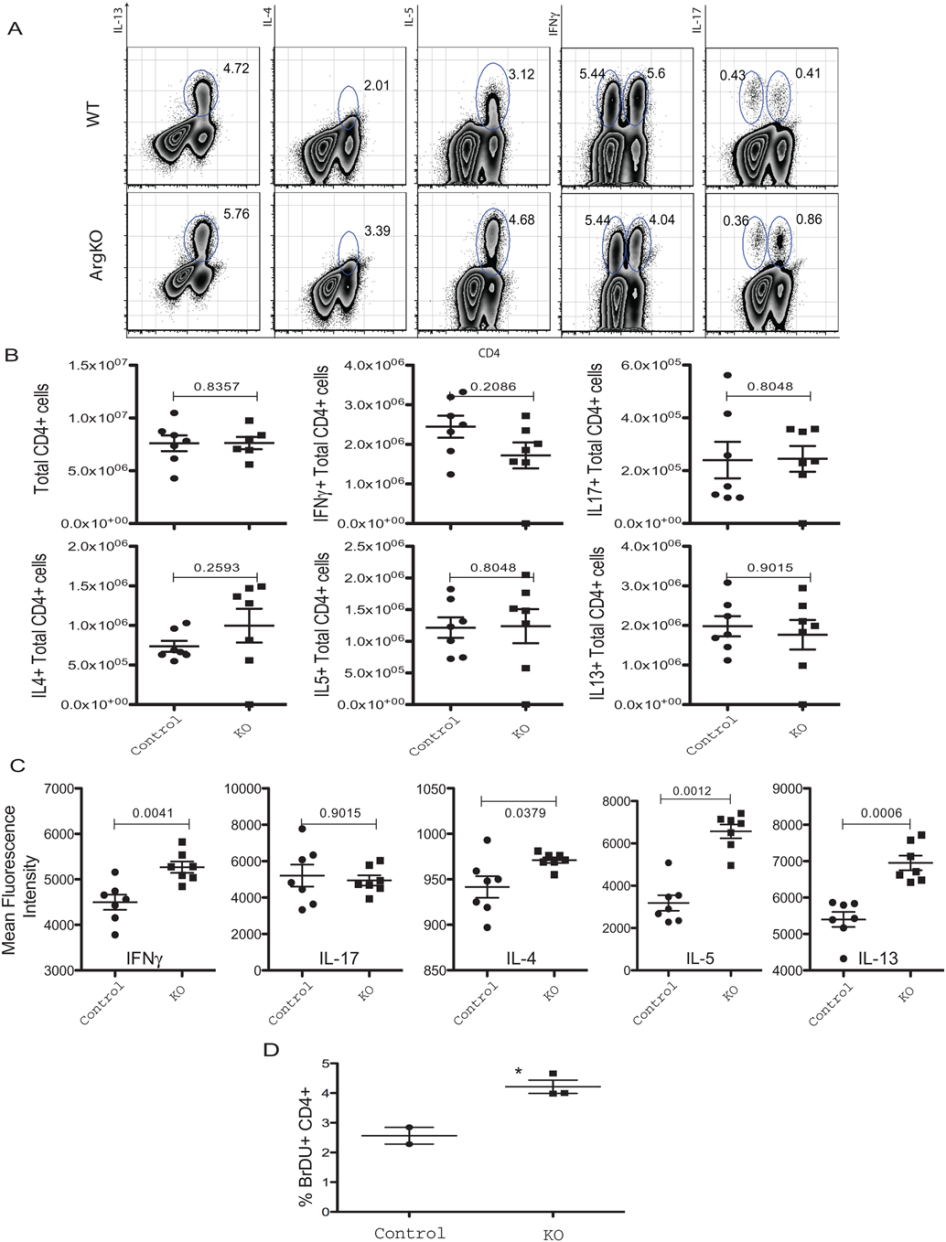
*Arg1*-expressing AAMs suppress Th2 cytokine production. Liver leukocytes isolated from 9-week *S. mansoni*–infected mice were separated, counted, and cultured with PMA, Ionomycin, and Brefeldin A for 3 hours and analyzed for ex-vivo cytokine production capability. (A) Representative FACS plots of IL-13, IL-4, IL-5, IFN-γ, and IL-17-producing CD4^+^ T cells. (B) Total number of cytokine-producing CD4^+^ cells in the liver leukocyte preparation (n = 7/group). (C) Magnitude of cytokine production by CD4^+^ cells displayed as geometric mean of fluorescence intensity. Statistically significant differences are shown (n = 7/group). (D) 9-week *S. mansoni*–infected mice were treated with BrdU for 3 days by i.p. injection prior to sacrifice, and then mesenteric lymph nodes cells were isolated and stained for CD4^+^ and BrdU incorporation (n = 3/group). All experiments were repeated twice with similar results.

Although AAMs were previously shown to protect mice from the highly lethal effects of Th1-type pathology [23], our data demonstrate that macrophages expressing Arg1 are required for the suppression of Th2-associated morbidity and mortality in chronic schistosomiasis. To identify a mechanism that could explain the increased inflammatory and fibrotic responses in *Arg1* ^−/flox^ *;LysMcre* mice, we first examined whether T regulatory cell function was altered in the granulomatous tissues as Tregs are thought to play an important role in the downregulation of egg-induced pathology during *S. mansoni* infection [9]. While significant numbers of both FoxP3^+^CD25^+^ and FoxP3^+^CD103^+^ cells were detected in the liver following infection, there were no significant differences noted in control and *Arg1* ^flox/flox^ *;LysMcre* mice (Figure S2). There was however a small but consistent increase in the number of CD4^+^CD44^+^CD25^+^ effector T cells in the *Arg1* ^−/flox^ *;LysMcre* mice. Therefore, to determine whether Th1, Th2, or Th17 cytokine responses were altered, lymphocytes were isolated from the granulomatous livers and intracellular cytokine staining was performed for IFN-γ, IL-17, IL-4, IL-5, and IL-13. While no significant differences were noted in the frequency of cytokine producing cells in the liver (Figure 6A and 6B), we detected a consistent and significant increase in the production of IL-4, IL-5, and IL-13 in CD4^+^ T cells isolated from *Arg1* ^−/flox^ *;LysMcre* mice (Figure 6C). A small increase in IFN-γ was also observed, while IL-17 was similar in both groups. We also detected increased levels of IL-5 in the serum of *Arg1* ^−/flox^ *;LysMcre* mice (data not shown). In addition, in vivo proliferation studies conducted with bromodeoxyuridine (BrdU) incorporation and flow cytometric analysis suggested that the *Arg1* ^−/flox^ *;LysMcre* mice CD4^+^ T cells were proliferating more than WT cells (Figure 6D). Together, these data indicated that the *Arg1* ^−/flox^ *;LysMcre* mice were developing exaggerated effector T cells responses, thus providing a mechanism for the enhanced Th2-dependent pathology.

**Figure 7:**
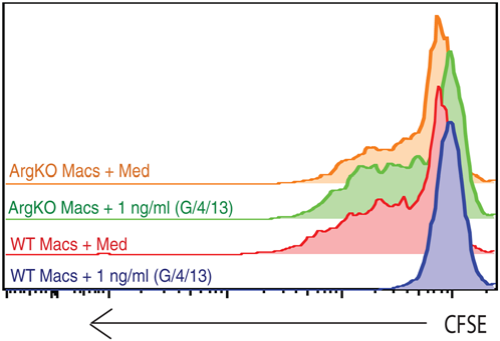
Arg1-expressing AAMs suppress T cell proliferation. Day-4 thioglycollate-elicited peritoneal macrophages from WT or *Arg1* ^−/flox^ *;LysMcre* mice were treated with 1 ng/ml of cytokine cocktail (IL4+IL13+GM-CSF) for 36 hours before addition of CFSE labeled LN cells from OT-II mice and cognate OVA peptide. Proliferation of CD4^+^ cells was examined at 96 hours of co-culture in RPMI+10% FCS. The experiment was repeated twice with similar results.

Because Th2 cytokines have been historically described as “anti-inflammatory” mediators [30], we investigated whether Arg1 was required by alternatively-activated macrophages to inhibit antigen specific T cell proliferation. For these studies, macrophages were prepared from both WT and *Arg1* ^−/flox^ *;LysMcre* mice and treated with a cocktail of IL-4/IL-13/GM-CSF for 36 hr to induce alternative activation before the addition of CFSE labeled lymph node cells isolated from OT-II mice and cognate OVA peptide. Strikingly, while untreated WT and KO macrophages induced robust T cell proliferation, WT cells pretreated with IL-4/IL-13/GM-CSF completely failed to stimulate T cell proliferation (Figure 7). In marked contrast, AAMs generated from *Arg1* ^−/flox^ *;LysMcre* mice behaved like untreated macrophages, indicating that Th2-induced Arg1 expression is required for the suppressive activity of AAMs. Next, we examined if IL-10 and TGF-β1 were required for the suppressive activity. For these studies macrophages were treated with increasing doses of IL-4, IL-13, and GM-CSF for 36 h prior to introduction of CSFE-labelled OVA-specific T cells. In some cultures, neutralizing mAbs to TGF-β1 or IL-10 were included either alone or in combination.

**Figure 8:**
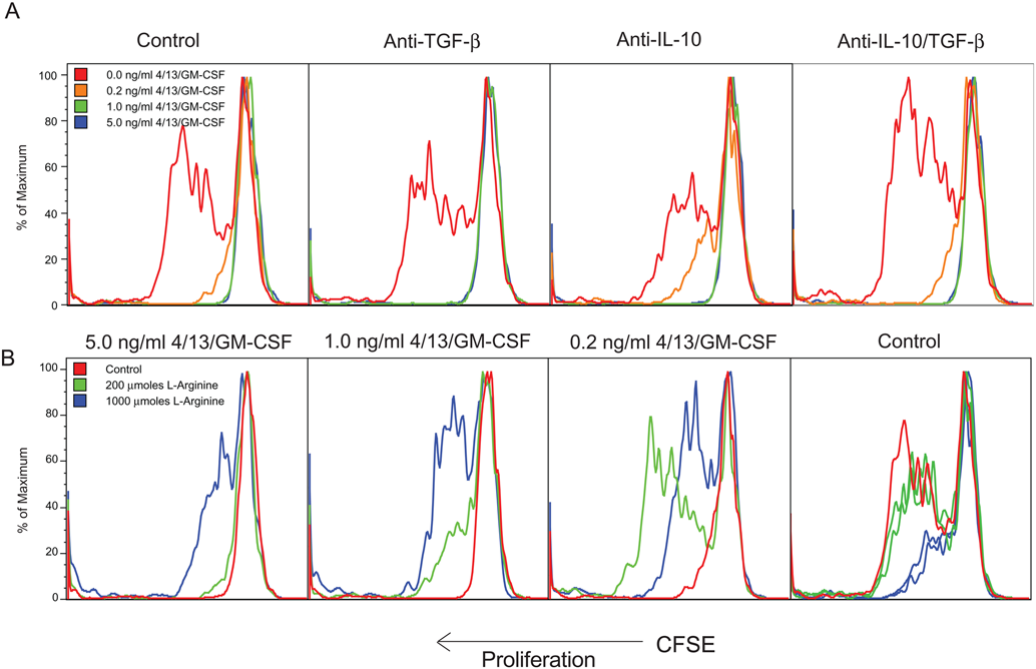
The suppressive activity of AAMs is IL-10 and TGF-β independent. (A) Day 4 peritoneal macrophages from thioglycollate-injected WT mice were treated with increasing doses of a cytokine cocktail of IL-4, IL-13, and GM-CSF for 36 hours before addition of CFSE-labeled LN cells from OT-II mice and cognate OVA peptide. Proliferation of CD4^+^ cells was examined at 96 hours of co-culture in RPMI+10% FCS. Some groups were also treated with anti-TGF-β (10 µg/ml), anti-IL-10 (10 µg/ml), or a combination of both mAbs when T cells were added to the macrophage APCs. (B) In a second series of experiments, the macrophage/T cell cultures were supplemented with L-arginine hydrochloride at the indicated concentrations, again at the time when T cells were added. All experiments were repeated three times with similar results.

While unstimulated macrophages induced robust T cell proliferation, as little as 0.2 ng/ml of IL-4/IL-13/GM-CSF was sufficient to inhibit proliferation by 80–90% (Figure 8A, red vs. orange). Higher doses inhibited T cell proliferation completely. Importantly, the introduction of neutralizing mAbs to IL-10 and TGF-β1 to the cultures had no effect. In parallel we also examined whether competition between AAMs and T cells for arginine might be responsible for the suppressive activity. When the cultures were supplemented with excess L-arginine, proliferation was restored (Figure 8B) and dependent on the dose of IL-4/IL-13/GM-CSF added to the cultures: when the macrophages were pre-treated with a high dose of IL-4/IL-13/GM-CSF (5 ng/ml) to induce alternative activation, large quantities of excess L-arginine (1000 µmoles) were required to restore CD4^+^ T cell proliferation (Figure 8B, left panel). In contrast, when lower doses of Th2 cytokines were used (1.0 or 0.2 ng/ml), less L-arginine was required (Figure 8B, middle panels). The addition of L-arginine alone had no effect on T cell proliferation when control macrophages were used, and it even exhibited an inhibitory effect when used had high doses (Figure 8B, right panel).

**Figure 9:**
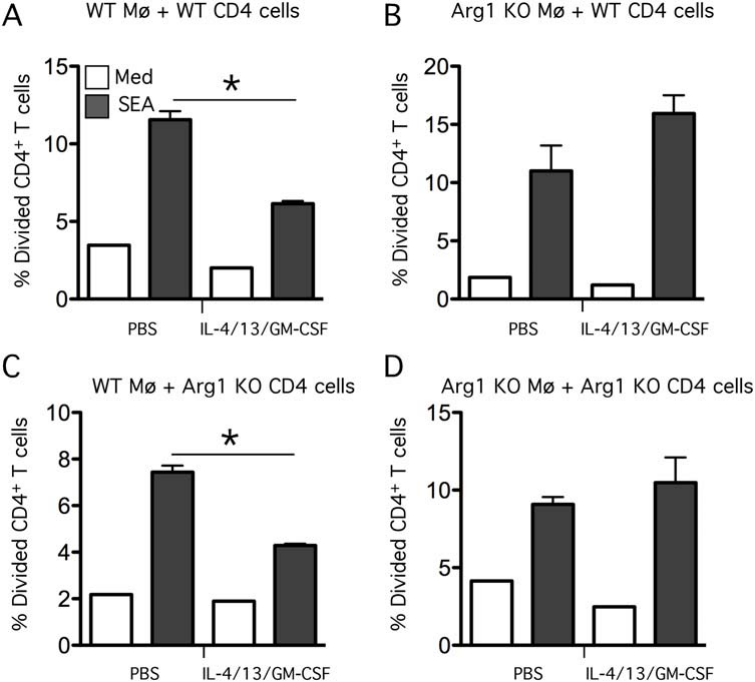
Arg1 is required to suppress schistosome-specific T cell proliferation. 2×10^5^ thioglycollate-elicited macrophages from (WT) control Arg1^flox/flox^ (A,C) and (KO) *Arg1* ^−/flox^ *;LysMcre* mice (B,D) were pre-treated with a cocktail of IL-4/IL-13/GM-CSF at a concentration of 1 ng/ml of each cytokine or PBS. CD4^+^ cells were isolated from the livers of 9-week *S. mansoni*–infected WT (A,B) or *Arg1* ^−/flox^ *;LysMcre* mice (C,D), labeled with CFSE, and then added to the macrophage cultures at a concentration of 1×10^5^ cells per well in RPMI+10% FCS. Wells were either left unstimulated (Med) or stimulated with 20 µg/ml of SEA. After 84 hours, proliferation was analyzed by flow cytometry and represented as the percentage of CD4 cells that had divided in each of the conditions. The experiment was conducted twice with similar results.

Finally, to determine whether T cells isolated from *S. mansoni* mice would respond in a similar manner, CD4^+^ T cells were isolated from infected mice and soluble egg antigens (SEA) were used as the antigenic stimulus. Macrophages isolated from either control *Arg1* ^flox/flox^ and *Arg1* ^−/flox^ *;LysMcre* mice were used as the APCs. Macrophages were either left unstimulated or pre-treated with IL-4, IL-13, and GM-CSF (Figure 9). Unstimulated WT macrophages stimulated robust T cell proliferation in response to SEA whether WT (Figure 9A) or KO (Figure 9C) CD4^+^ T cells were employed. In contrast, when WT macrophages were pretreated with IL-4/13/GM-CSF, SEA-specific T cell proliferation was reduced (Figure 9A and 9C), thus supporting our earlier studies with OVA-specific T cells (Figure 8). However, when *Arg1* ^−/flox^ *;LysMcre* macrophages were used as APCs, the pre-treatment with IL-4/13/GM-CSF was not inhibitory (Figure 9B and 9D). Thus, these data suggest that AAMs were depleting the available stores of arginine, which is required for T cells to proliferate when stimulated with SEA. This mechanism may explain in part why the *Arg1* ^−/flox^ *;LysMcre* developed severe CD4^+^ Th2-associated pathology.

## Discussion

As with most helminth infections, schistosomiasis is associated with the development of a CD4^+^ Th2 cell response [9]. Pearce and colleagues showed that survival during acute *S. mansoni* infection is highly dependent on IL-4 signaling [22],[28]. In the absence of IL-4, C57BL/6 mice exhibit severe cachexia and rapidly succumb to *S. mansoni* infection. They concluded that the morbidity was due to increased TNF-α and nitric oxide production in the affected tissues. Studies with *S. mansoni* infected *Il4* ^−/−^ and *Il4* ^−/−^;*Il13* ^−/−^ mice suggested that in addition to developing hepatotoxicity, the integrity of the gut epithelium is compromised, leading to significant inflammation in the ileum, systemic leakage of intestinal contents, and development of lethal endotoxemia [31],[32]. Plasma levels of LPS also increased nearly 20-fold and recruitment of eosinophils was dramatically reduced in the intestines, confirming that the acute mortality of *Il4* ^−/−^ mice was due to the increased Th1/NOS2/endotoxemia and reduced Th2 effector response. Similar to *Il4* ^−/−^, *Il4* ^−/−^;*Il13* ^−/−^, and *Il4ra* ^−/−^ mice [22],[29],[31],[32], mice that are genetically deficient in AAMs (*Il4ra* ^−/flox^;*LysMcre*) displayed 100% mortality when acutely infected with *S. mansoni* [23]. Here again, death was associated with increased Th1 cytokine production, hepatic and intestinal histopathology, increased NOS2 activity, elevated liver enzymes, and endotoxemia. The absence of AAMs in these mice, however, had no effect on the development of fibrosis. Based on these observations, the authors concluded that IL-4Rα signaling in AAMs is critical for host survival in acute *S. mansoni* infection [23]. These findings were important because they suggested that the protective activity of the Th2 response in schistosomiasis is directly linked to its role in promoting the differentiation of AAMs.

Because hepatic fibrosis, portal hypertension, and variceal bleeding are the primary causes of morbidity and mortality in human schistosomiasis [33], our research has focused on understanding the mechanisms regulating the development of chronic Th2-associated pathology [2]. Indeed, when mice are persistently infected with *S. mansoni*, the chronic Th2 response itself becomes highly pathogenic [34]. Previous studies with various cytokine deficient mice demonstrated that Th2 cytokines are critical to the development of hepatic fibrosis, portal hypertension, bleeding from collateral vessels, and ultimately death in schistosomiasis [10], [31], [35]–[37]. In chronically infected mice, morbidity and mortality primarily results from fibrosis-associated pathology instead of the “Th1 cytokine storm” and resulting endotoxic/hepatotoxic reaction that is observed in *Il4* ^−/−^, *Il4* ^−/−^;*Il10* ^−/−^, *Il4ra* ^−/−^, and *Il4ra* ^−/flox^;*LysMcre* mice [33]. In support of this conclusion, mice that are deficient in either IL-13 or IL-13Rα1 are protected from fibrosis and they survive significantly longer than chronically infected WT mice [31],[38],[39]. Consequently, depending on the duration and magnitude of the infection, Th2 cytokines exhibit both protective (IL-4, acute infections) and pathogenic (IL-13, chronic infections) activity in the mouse model of schistosomiasis [40],[41]. Surprisingly, the development of Th2-driven fibrosis appeared normal in *S. mansoni* infected AAM-deficient *Il4ra* ^−/flox^;*LysMcre* mice [23]. Consequently, Herbert and colleagues concluded that AAMs are not required for Th2-dependent granuloma formation or fibrosis in schistosomiasis. Nevertheless, because 100% of the infected *Il4ra* ^−/flox^;*LysMcre* mice died at the acute stage of infection, it was impossible to evaluate the role of AAMs in chronic schistosomiasis using these mice.

In contrast to *Il4ra* ^−/flox^;*LysMcre* mice, AAMs develop normally in *Arg1* ^−/flox^ *;LysMcre* mice; however, their macrophages are deficient in the key gene that differentiates AAMs from other macrophage subpopulations [1]. Thus, by specifically targeting Arg1 in macrophages, our conditional knockout mice provided an ideal tool to dissect the role of AAMs in chronic schistosomiasis. Strikingly, in contrast to the findings with *Il4ra* ^−/flox^;*LysMcre* mice, our data suggest that Arg1-expressing AAMs are not required to suppress acute Th1/NOS2/LPS-mediated morbidity and mortality in schistosomiasis. Indeed, we obtained no evidence that the *Arg1* ^−/flox^ *;LysMcre* mice were developing lethal endotoxemia or hepatotoxicity during acute schistosome infection. Concordant results were obtained with Arg1^flox/flox^;*Tie2cre* mice, which we showed have an almost complete deletion of Arg1 in all macrophage types examined [8]. Instead, our data suggest that the major function of Arg1 is to downmodulate granulomatous inflammation in the liver and intestine and to slow the progression of Th2-dependent fibrosis in chronically infected mice. As such, our studies identified a novel role for AAMs in the pathogenesis of schistosomiasis. These results also illustrated the importance of investigating the specific contributions of the unique genes that characterize AAMs [19],[42], in addition to analyzing the function of AAMs as a whole [13],[15],[23],[43].

Th2-activated macrophages have had long history of being described as ‘suppressor’ cells [30]. Nevertheless, most of these studies have concentrated on the role of myeloid-derived suppressor cells (MDSCs) in cross-regulating type-1 immunity and inhibiting the function of classically activated macrophages [24], [44]–[50]. Indeed, the role of Arg1-expressing macrophages in the regulation of Th2 responses is much less clear. Fortunately, the *Arg1* ^−/flox^ *;LysMcre* mice provided an ideal model system to formally test the activity of macrophage-derived Arg1 in an important Th2 disease model. The results from our studies demonstrate that IL-4/IL-13-activated macrophages potently inhibit T cell proliferation. T cell proliferation was restored when Arg1 activity was deleted in AAMs or when WT AAMs were provided exogenous arginine, illustrating a critical role for arginine depletion in the suppressive activity of Arg1^+^ AAMs [24]. No significant contribution for IL-10 or TGF-β1 was observed in these studies, suggesting that arginine depletion by Arg1-expressing macrophages was the primary suppressive mechanism. In agreement with these observations, the Th2 cytokine response was enhanced in the infected *Arg1* ^−/flox^ *;LysMcre* mice. Moreover, BrdU incorporation studies confirmed a suppressive role for Arg1 in vivo. Thus, these studies demonstrated unequivocally that Arg1-expressing AAMs function as potent inhibitors of chronic Th2 responses [24],[48]. Several previous studies suggested that AAMs promote the development of CD4^+^ Th2 cell responses in vivo [51]–[53]. Nevertheless, not only were Arg1 expressing macrophages not required for the development of Th2 responses or fibrosis, our data showed that they inhibit these responses. Therefore, it will be important to determine whether this macrophage-associated Arg1-dependent mechanism is operating in other Th2-associated diseases [21]. It will also be important to determine whether the depletion of L-arginine by alternatively-activated macrophages in specific microenvironments like granulomas or tumors contributes to the local suppression of T cell responses and/or myofibroblast activity *in vivo*.

While macrophage-specific Arg1 was originally hypothesized to be an inducer of Th2-driven pathology [1],[2],[43],[54], the studies conducted here with *Arg1* ^−/flox^ *;LysMcre* and *Arg1* ^flox/flox^ *;Tie2cre* mice revealed an unanticipated function for the Th2-inducible Arg1 isoform. Instead of promoting Th2 disease, our data suggest that macrophage-specific Arg1 contributes to the resolution of schistosomiasis by inhibiting CD4^+^ T cell effector function. It is also possible that the increased collagen deposition in the knockout mice results from changes in competition for L-arginine by the cytosolic and mitochondrial arginase isoforms. The net arginine-to-proline conversion could be higher in Arg1 null macrophages than in WT macrophages because of differential subcellular compartmentation of ornithine-metabolizing enzymes, which function downstream of the arginases. For example, the cytosolic Arg1 in WT macrophages may compete for substrate with the mitochondrial Arg2, thus diverting ornithine preferentially to a different metabolic fate than ornithine produced by Arg2 within mitochondria. Consequently, the metabolic fate of ornithine in Arg1 null macrophages could be shifted dramatically from the fate observed in WT macrophages. Finally, it is also possible that Arg1-expressing macrophages are simply competing with fibroblasts for L-arginine and in the absence of macrophage-specific Arg1, more substrate is available to activated myofibroblasts, resulting in increased collagen production. Indeed, all three of these mechanisms could be contributing the exacerbated fibrosis observed in the infected macrophage-specific Arg1 null mice.

Recently, we showed that Arg1-expressing macrophages also regulate type-1 responses [8]. However, in contrast to diseases dominated by Th2-type cytokines in which Arg1 expression is greatly increased by IL-4 and 13 signaling through the transcription factor STAT6 [11],[55], during Th1 responses a novel TLR-dependent but STAT6-independent pathway of Arg1 expression was identified [8]. In this case, the specific elimination of Arg1 increased production of the anti-microbial mediator NO, which favored host survival during *T. gondii* infection and decreased lung bacterial loads during tuberculosis. Therefore, although macrophage Arg1 expression is more commonly associated with AAMs found in helminth infections and allergic reactions, we discovered that CAMs also produce Arg1. The combined results from both studies suggest that macrophage-derived Arg1 exhibits important but distinct regulatory activities during Th1 and Th2-type immune responses. During Th1 responses, CAM-derived Arg1 antagonizes NOS2 activity, which promotes infection by intracellular pathogens, while in Th2 responses Arg1-expressing AAMs contribute to the resolution of chronic Th2-driven inflammation and fibrosis by functioning as suppressor cells.

## Methods

### Ethics statement

All animal work was conducted according to relevant national and international guidelines.

### Mice, parasite infections

The generation of control Arg1^flox/flox^, C57BL/6 *Arg1* ^−/flox^ *;LysMcre* , and BALB/c and C57BL/6 *Arg1* ^flox/flox^ *;Tie2cre* mice have been described [8]. All mice were bred and housed under specific pathogen-free conditions at the National Institutes of Health in an American Association for the Accreditation of Laboratory Animal Care approved facility. The NIAID animal care and use committee approved all experimental procedures. For infections, mice were percutaneously exposed via the tail with 30–35 cercariae of a Puerto Rican Strain of *S. mansoni* (NMRI) that were obtained from infected *Biomphalaria glabrata* snails (Biomedical Research Institute, Rockville, MD). All animals underwent perfusion at the time of sacrifice so that worm and tissue egg burdens could be determined.

### Histopathology and fibrosis

Pulmonary and hepatic granuloma measurements were determined from histological sections stained with Wright's Giemsa or Picrosirius Red (Histopath of America, Clinton, MD). Thirty granulomas per mouse were included in all analyses. An experienced pathologist evaluated the percentages of small and large lymphocytes, macrophages, fibroblasts, and eosinophils in the same sections. The number of schistosome eggs in the liver and the gut and the collagen content of the liver, as measured by hydroxyproline levels, were determined as previously described [11]. Hepatic collagen was measured after hydrolysis of a 200-mg portion of liver in 5 ml of 6N HCl at 110°C for 18 h. The increase in hepatic hydroxyproline was positively related to egg numbers in all experiments and hepatic collagen is reported as the increase above normal liver collagen in micromoles per 10,000 eggs; (infected liver collagen – normal liver collagen)/liver eggs ×10^−4^ or micromoles per worm pair. At late chronic time points, fibrosis is reported as total liver collagen per liver. The same individual scored all histological features and had no knowledge of the experimental design.

### Aminoguanidine treatments

Mice were given a 2.5% solution of AG hemisulfate (Sigma, St. Louis, Mo.) in sterilized drinking water on week 6–12 following *S. mansoni* exposure [26],[27]. The dose employed was shown previously to completely suppress nitric oxide production in infected mice [56].

### Peritoneal macrophages

Peritoneal macrophages were elicited by injecting 3ml of autoclaved thioglycollate broth i.p/mouse. Macrophages were harvested 96 hours post i.p injection.

### Nitrite analysis and arginase assays

Arginase assays were previously described [10]. Briefly, neutrophils were plated at 5×10^5^ per well directly ex vivo in 96 well tissue culture plates, macrophages were plated at a similar density and stimulated with combinations of IL-4, IL-13, and IL-21 (20ng/mL). IL-21 was added 6 hours prior to IL-4 or IL-13 stimulation. Following stimulation, cells were washed with PBS and lysed with 0.1% Triton X-100 containing protease inhibitor (Roche, Nutley, NJ). Lysates were transferred into a 96 well PCR plate and incubated with 10mM MnCl_2_ and 50mM Tris HCl (pH 7.5) for 10 min at 55°C. 25ul of lysate was removed and added to 25 µl 1M arginine (pH 9.7) in a new PCR plate and incubated for 1–2 hours at 37°C. 5 µl of each sample was added in duplicate to a 96 well ELISA plate along with 5 µl of each standard, diluted in same assay conditions, starting at 100 mg/dL. Urea determination reagent from BioAssay Systems Quantichrome Urea Assay Kit (Bioassay Systems, Hayward, CA) was used according to the manufacturer's protocol. The concentration of nitrite in supernatants of thioglycollate elicited macrophages was determined spectrophotometrically using Griess reagent. Macrophages were plated at 5×10^5^ per well in 96 well tissue culture plates and stimulated with combinations of IL-4 (20ng/mL), IL-13 (20ng/mL), IL-21 (20ng/mL), IFN-γ (100units/mL), and LPS (100ng/mL). Supernatants were collected after 24 hours, mixed 1/1 with Griess reagent, and absorbance measured at 543 nm using a SpectraMax 190 (Molecular Devices, Sunnyvale, CA). The nitrite concentration was determined using sodium nitrite 22 as a standard.

### RNA isolation and purification and real time polymerase chain reaction

Thioglycollate elicited macrophages were lysed with Buffer RLT (Qiagen Sciences, Valencia, CA), containing 2-mercaptoethanol, and spun through Qiashredder tubes (Qiagen Sciences, Valencia, CA). Total RNA was extracted using the RNeasy Mini Kit from Qiagen (Qiagen Sciences, Valencia, CA). Individual sample RNA (0.1 µg) was reverse-transcribed using Superscript II (Invitrogen, Carlsbad, CA) and a mixture of oligo (dT) and random primers. Real- time polymerase chain reaction (RT-PCR) was performed on an ABI Prism 7900 sequence detection system (Applied Biosystems, Foster City, CA). Relative quantities of mRNA for several genes was determined using SYBR Green PCR Master Mix (Applied Biosystems, Foster City, CA) and by the comparative threshold cycle method as described by Applied Biosystems for the ABI Prism 7700/7900 sequence detection systems. In this method, mRNAs for each sample were normalized to hypoxanthine guanine phosphoribosyl transferase (HPRT) mRNA amounts and then expressed as a relative increase or decrease compared with uninfected controls. Sequences for HPRT, *Mrc1* (mannose receptor), and *Chi3l3* (Ym1) were published previously [39].

### Flow cytometry and ICC

Roughly 200 mg of granulomatous liver tissue was disrupted into a single cell suspension by grinding through a 70 µm nylon mesh. The WBCs from liver cells were separated on a 34% Percoll gradient (350×g for 20 min) (Sigma-Aldrich, St. Louis, MO). MLN and liver WBC's were treated with 2 ml of ACK lysis buffer (Quality Biological, Gaithersburg, MD) for 2 min. ICC: Liver leukocytes were stimulated with PMA (10ng/ml), Ionomycin (1ug/ml) and BFA (10ug/ml) (Sigma) for 3 hrs. Cells were surface stained for CD4 APC (BD Biosciences, San Jose, CA), fixed in 2% formaldehyde for 20 min at RT, permeabolized with 0.1% saponin buffer (Sigma-Aldrich, St. Louis, MO) and stained for IL-13 FITC (Centocor, Wayne, PA) and acquired with FACS Calibur (BD Biosciences, San Jose, CA). Data were analyzed using Flowjo V7.2.

### Microscopy

Portions of livers from control and *Arg1* ^−/flox^ *;LysMcre S. mansoni* infected mice were removed and frozen in liquid nitrogen. Cryostat-cut tissue sections (8 µm) were fixed in acetone and stained with the following reagents: F4/80 Alexa 647 (Invitrogen, Carlsbad, CA), and VECTASHIELD HardSet Mounting Medium with DAPI (Vector Laboratories, Burlingame, CA). Slides from weeks 9, 12, and 22 post infection were analyzed for collagen content via second harmonic emission. Second harmonic emission was generated using a Spectra Physics MaiTai HP laser tuned to 810nm (Spectra Physics Corp., CA, USA). Image acquisition was performed on a Leica SP2 confocal microscope (Leica Microsystems, Exton, PA USA) using a 20× oil immersion objective NA 0.70, and a dry condenser, NA. 0.9. The forward propagating signal was detected using the transmitted light detector. To isolate the SHG signal et405/20 and SP700nd8 filters were inserted in the light path between the condenser and detector. (Chroma Technologies Corporation, Rockingham, VT). The SHG intensity was detected without polarization selection. Mean fluorescence intensity was recorded for each individual slide and averaged for each group as a measure for the presence of collagen surrounding an individual granuloma.

### Endotoxin Measurement

Serum endotoxin was determined using an Endochrome™ limulus amebocyte lysate kit (Charles River Laboratories, Wilmington, MA) per the manufacturer's specifications.

### BrdU proliferation assays and flow cytometry

On approximately wk 8.5 post-infection, groups of WT and *Arg1* ^−/flox^ *;LysMcre* mice were injected with 100 µl BrdU (BD Pharmingen) at 10mg/ml in sterile PBS for 3 consecutive days prior to sacrifice. Mesenteric lymph node cells were isolated and the BrdU flow kit staining protocol provided by Pharmingen was followed to assess BrdU incorporation and cellular proliferation.

### Ex vivo intracellular cytokine staining

Leukocytes isolated from granuloma or mesenteric lymph nodes were stimulated with PMA (10 ng/ml), Ionomycin (1 µg/ml) and BFA (10 µg/ml) for 3 hrs. Cells were surface stained with PE-Cy5 conjugated αCD4, fixed in 2% formaldehyde for 20 min. at room temperature, permeabolized with 0.1% saponin buffer for 30 min. and further stained with FITC conjugated α-IFN-γ, Phycoerythrin conjugated α-IL-13 (Centocor), Alexa-647 conjugated α-IL-4 and Allophycocanin conjugated α-IL-5, or conjugated α-IL-17 before acquiring with FACS Calibur. Antibodies were from BD Pharmingen except mentioned otherwise. Data were analyzed in Flowjo V8.

### Statistics

Hepatic fibrosis (adjusted for egg number) decreases with increasing intensity of infection (worm pairs). Therefore, these variables were compared by analysis of covariance, using the logarithm of total liver eggs as the covariate and the logarithm of hydroxyproline content per egg. All other data was analyzed with Prism (Version 5; GraphPad). Data were considered statistically significant for *P* values less than 0.05, obtained with a two-tailed *t*-test.

## Supporting Information

Figure S1 Larger granulomas form in the liver and gut of *Arg1-/flox;lysMcre* mice. Control and *Arg1-/flox;lysMcre* mice were infected with 35 *S. mansoni* cercariae and sacrificed on week 9 post-infection. Individual mice were assessed (n = 7, both groups) for granuloma volume microscopically and reported as average granuloma volume+SEM. The granulomas in the gut and liver were significantly larger in *Arg1flox/flox;lysMcre* mice, p<0.001.
(0.20 MB PDF)

Figure S2 Staining of various activation markers in liver leukocytes. Liver leukocytes isolated from 9-week *S. mansoni*-infected WT and *Arg1-/flox;lysMcre* mice were separated, counted, and stained for the various markers shown in the figure. Significant differences are noted in the figure.
(0.57 MB PDF)

Table S1 *Schistosoma mansoni* infection in control and *Arg1-/flox;lysMcre* mice. Control and *Arg1-/flox;lysMcre* mice were infected with 35 *S. mansoni* cercariae and sacrificed on weeks 9, 12, and 22 post-infection. Worm numbers and tissue egg burdens enumerated as described in Methods, are presented as mean±s.e.m. A minimum of 10 mice were used in each group. All experiments were repeated at least two times.
(0.02 MB PDF)
