# Supplementary figures and images for: Arginase-1–Expressing Macrophages Suppress Th2 Cytokine–Driven Inflammation and Fibrosis
Source: PLoS Pathog. 2009 Apr 10;5(4):e1000371. doi: 10.1371/journal.ppat.1000371 (PMC2660425; doi:10.1371/journal.ppat.1000371)

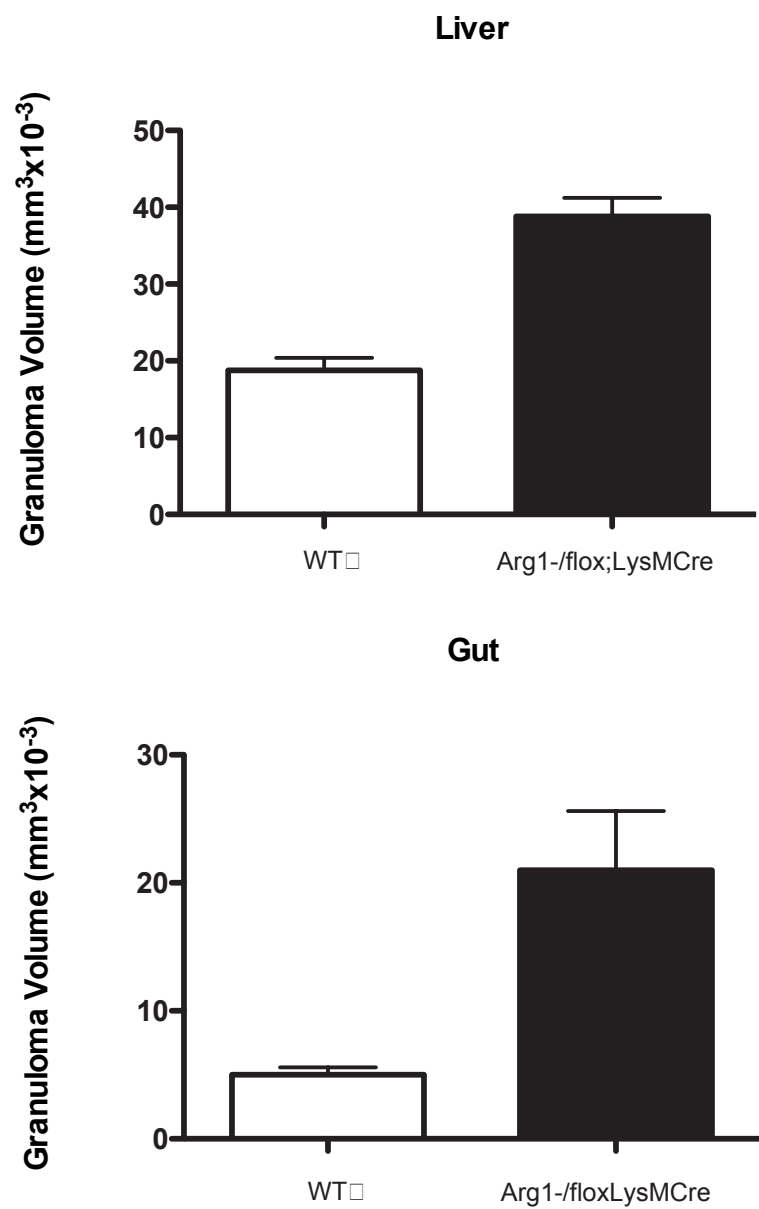

Supplement: Figure S1 — Larger granulomas form in the liver and gut of Arg1-/flox;lysMcre mice. Control and Arg1-/flox;lysMcre mice were infected with 35 S. mansoni cercariae and sacrificed on week 9 post-infection. Individual mice were assessed (n = 7, both groups) for granuloma volume microscopically and reported as average granuloma volume+SEM. The granulomas in the gut and liver were significantly larger in Arg1flox/flox;lysMcre mice, p<0.001. (0.20 MB PDF) [file ppat.1000371.s001.pdf]

CD4+ Regulatory T cell subsets

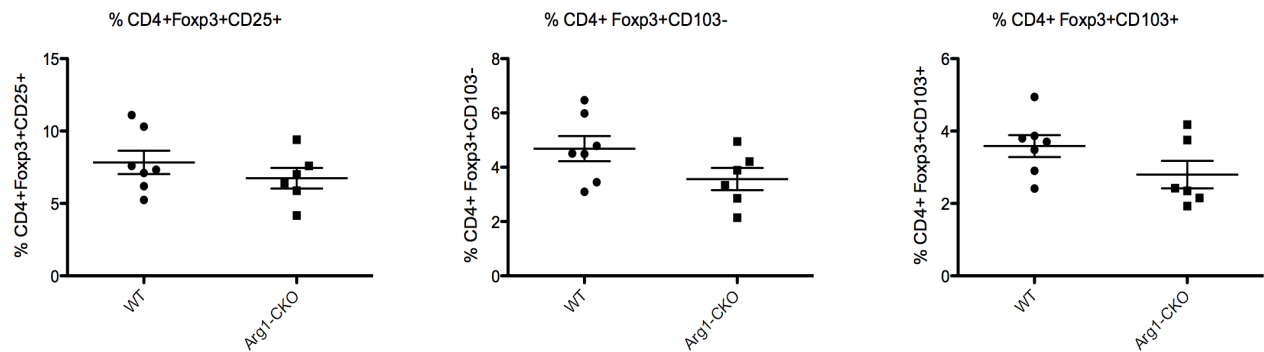

CD4+ Effector T cells

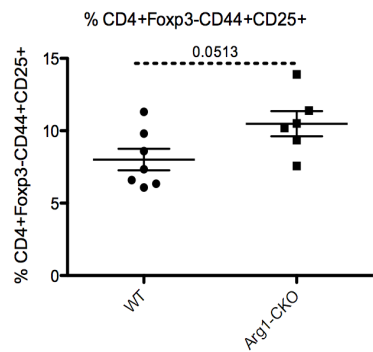

CD8+ Effector T cells

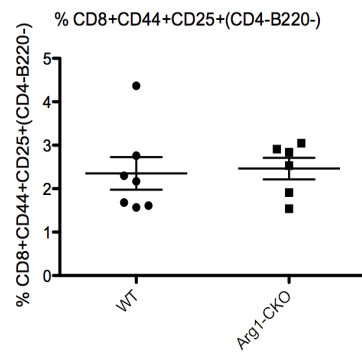

Supplement: Figure S2 — Staining of various activation markers in liver leukocytes. Liver leukocytes isolated from 9-week S. mansoni-infected WT and Arg1-/flox;lysMcre mice were separated, counted, and stained for the various markers shown in the figure. Significant differences are noted in the figure. (0.57 MB PDF) [file ppat.1000371.s002.pdf]
