# Supplementary material for: Arginase-1–Expressing Macrophages Suppress Th2 Cytokine–Driven Inflammation and Fibrosis
Source: PLoS Pathog. 2009 Apr 10;5(4):e1000371. doi: 10.1371/journal.ppat.1000371 (PMC2660425; doi:10.1371/journal.ppat.1000371)
Supplement: Table S1 — Schistosoma mansoni infection in control and Arg1-/flox;lysMcre mice. Control and Arg1-/flox;lysMcre mice were infected with 35 S. mansoni cercariae and sacrificed on weeks 9, 12, and 22 post-infection. Worm numbers and tissue egg burdens enumerated as described in Methods, are presented as mean±s.e.m. A minimum of 10 mice were used in each group. All experiments were repeated at least two times. (0.02 MB PDF) [file ppat.1000371.s003.pdf]

Supplemental Table 1-- Parasite Burden for control and Arg1$^{-/flox}$;lysMcre mice after 9, 12, and 22 weeks of infection with S. mansoni

		Worm Pairs (Ave+SEM)	Eggs/Worm Pair (Thousands)	Liver Eggs (1000s)	Gut Eggs (1000s)
Week 9	Control	5.00±0.77	5.30±0.71	17.25±3.03	5.99±1.06
	Arg1$^{-/flox}$;lysMcre	3.22±0.49	4.60±0.46	9.92±1.25	3.53±0.63
Week 12	Control	2.37±0.27	13.99±1.17	55.90±8.40	2.99±0.91
	Arg1$^{-/flox}$;lysMcre	3.43±0.61	14.99±0.77	37.70±6.40	3.33±1.05
Week 22	Control	3.00±0.42	22.54±5.19	49.70±4.60	ND
	Arg1$^{-/flox}$;lysMcre	2.90±0.36	25.70±4.10	63.60±9.70	ND

ND - Not determined
